# Trimethyl Chitosan Hydrogel Nanoparticles for Progesterone Delivery in Neurodegenerative Disorders †

**DOI:** 10.3390/pharmaceutics11120657

**Published:** 2019-12-06

**Authors:** Maria Cristina Cardia, Anna Rosa Carta, Pierluigi Caboni, Anna Maria Maccioni, Sara Erbì, Laura Boi, Maria Cristina Meloni, Francesco Lai, Chiara Sinico

**Affiliations:** 1 Department of Life and Environmental Sciences, Unit of Drug Sciences, University of Cagliari, 09124 Cagliari, Italy; cardiamr@unica.it (M.C.C.); caboni@unica.it (P.C.); maccion@unica.it (A.M.M.); erbisara@gmail.com (S.E.); mariacristina.meloni@unica.it (M.C.M.); sinico@unica.it (C.S.); 2 Department of Biomedical Sciences, University of Cagliari, 09124 Cagliari, Italy; acarta@unica.it (A.R.C.); laurettaboi@hotmail.it (L.B.)

**Keywords:** trimethyl chitosan, progesterone, brain, hydrogel nanoparticles

## Abstract

Progesterone is a sex hormone which shows neuroprotective effects in different neurodegenerative disorders, including Parkinson’s disease, stroke, and Alzheimer’s disease. However, the pharmacokinetic limitations associated with the peripheral administration of this molecule highlight the need for more efficient delivery approaches to increase brain progesterone levels. Since the nose-to-brain administration of mucoadhesive hydrogel nanoparticles is a non-invasive and convenient strategy for the delivery of therapeutics to the central nervous system, in this work, progesterone-loaded hydrogel nanoparticle formulations have been prepared, characterized, and tested in vivo. Nanoparticles, loaded with different progesterone concentrations, have been obtained by polyelectrolyte complex formation between trimethyl chitosan and sodium alginate, followed by ionotropic gelation with sodium tripolyphosphate as a cross-linking agent. All formulations showed a mean diameter ranging from 200 nm to 236 nm, a polydispersity index smaller than 0.23, and a high progesterone encapsulation efficiency (83–95%). The zeta potential values were all positive and greater than 28 mV, thus ensuring nanoparticles stability against aggregation phenomena as well as interaction with negative sialic residues of the nasal mucosa. Finally, in vivo studies on Sprague–Dawley male rats demonstrated a 5-fold increase in brain progesterone concentrations compared to basal progesterone level after 30 min of hydrogel nanoparticle inhalation.

## 1. Introduction

In recent years, research has focused on the potential repurposing of sex hormones, particularly progesterone (PG), for the treatment of neurodegenerative disorders, including Parkinson’s disease (PD), stroke, and Alzheimer’s disease [1,2]. Use of PG in PD initially raised interest following evidence of the higher incidence in men than in women [3,4]. PD is a complex pathology comprising multiple pathological events; therefore, molecules with several mechanisms of action, such as PG, are valuable options as neuroprotective agents [5]. Preclinical studies have suggested a neuroprotective effect of low doses of PG administered in several models of PD [6,7]. Similarly, the neuroprotective action of PG was also found in models of traumatic brain injury in both human and experimental animals [8,9,10] and is under investigation for cognitive impairment in Alzheimer’s disease [11]. Therefore, while current evidence points to PG as a valid and promising therapeutic approach for several CNS disorders, the pharmacokinetic limitations associated with the peripheral administration of this molecule highlight a need for more efficient delivery approaches to increase its brain levels.

In particular, oral administration of PG is characterized by low bioavailability. Indeed, its rapid absorption is associated with an elevated clearance rate due to intestine and first-pass metabolism [12,13]. Moreover, PG absorption is influenced by food intake, formulation excipients, and drug crystal diameter [14]. Several PG derivatives with a higher stability have been synthetized, but their use often relates to different undesirable effects, such as a decrease in total high-density lipoprotein cholesterol and an increased risk of fetal malformations [12,14]. For these reasons, in order to overcome low oral bioavailability, parenteral PG administration routes have been explored. Intravenous and intramuscular administrations determine high serum PG concentrations [12,13]; however, in the case of chronic therapy, daily injections can be uncomfortable and are characterized by low patient compliance.

Therefore, as several high-molecular-weight drugs have shown promising results in the treatment of CNS disorders, alternative routes for brain delivery are steadily investigated [15,16]. Nose-to-brain administration is a non-invasive and convenient strategy for the delivery of therapeutics to the CNS, bypassing the blood-brain barrier (BBB). Indeed, the nasal cavity can be employed not only for local administration, but also for the systemic delivery of different therapeutic agents (e.g., peptides, proteins, stem cells, etc.) due to its large surface area and high degree of vascularization. Drug administered by this route can enter the brain principally using olfactory or trigeminal nerve pathways. Moreover, other pathways involving vasculature, cerebrospinal fluid, and the lymphatic system have been individuated [17,18].

Even though it is often not easy to identify the exact pathways that an intranasally administered molecule follows to penetrate the CNS, it has been observed that different experimental factors can influence the amount and rate of brain accumulation. Usually, an increase in the drug contact time (residence time) with the nasal mucosa enhances the overall nose-to-brain drug delivery. In particular, it has been demonstrated that the use of mucoadhesive hydrogel nanoparticles (NPs) can increase the residence time and, consequently, the brain bioavailability of loaded drugs [19,20].

*N*,*N*,*N*-Trimethyl chitosan (TMC) is a hydrophilic polymer obtained by chitosan methylation which is able to form a stable gel in presence of sodium alginate (SA), an anionic water-soluble polysaccharide composed of alternating blocks of a-l-guluronic and b-d-mannuronic acid (b 1–4 linked) [21]. Similarly to chitosan, it is characterized by low toxicity, good biodegradability, and biocompatibility. However, compared to chitosan, TMC shows a higher positive charge and consequently, good solubility at all physiological pH values. Moreover, TMC shows excellent mucoadhesive properties as well as the ability to improve the paracellular permeation of hydrophilic compounds by means of interactions with the tight junctions [22,23].

With the aim of both identifying a more effective route for PG administration and clarifying whether the mucoadhesive properties of TMC can affect its brain accumulation, in this work we prepared PG-loaded TMC hydrogel NPs for PG brain delivery.

PG-loaded hydrogel NPs were prepared by polyelectrolyte complex formation between TMC and SA followed by ionotropic gelation with sodium tripolyphosphate (TPP) as a cross-linking agent. NPs were characterized in terms of size, size distribution, surface charge, and encapsulation efficiency. Moreover, NP stability and in vitro PG release were assessed. Finally, brain PG accumulation was evaluated in vivo on Sprague–Dawley male rats after PG hydrogel NP inhalation.

## 2. Materials and Methods

### 2.1. Materials

Low-molecular-weight chitosan, sodium iodide, *N*-methyl-pyrrolidinone, methyl iodide, sodium chloride, sodium hydroxide, sodium alginate, sodium tripolyphosphate, and deuterated PG (D9-PG) were purchased from Sigma Aldrich. PG was purchased from Galeno (Carmignano, Italy). For the in vivo experiments Sprague–Dawley rats were purchased from Envigo (Huntingdon, England, UK).

### 2.2. TMC Synthesis and Characterization

The synthesis of TMC was performed starting with low-molecular-weight chitosan, slightly revising already reported procedures [21]. Chitosan (2 g) and sodium iodide (4.8 g) were dispersed in *N*-methylpyrrolidinone (80 mL) into a three empty flasks immersed in a water bath. The suspension was stirred vigorously with a paddle stirrer at a controlled temperature (60 °C) for 20 min. After stabilization of the temperature, an NaOH solution (11 mL, 15% *w/w*) and methyl iodide (12 mL) were added to the mixture, keeping the temperature at 60 °C (± 5 °C) for 60 min. Then, methyl iodide (5 mL) and NaOH solution (10 mL, 15% *w/w*) were added again to the mixture, and the suspension was stirred at 60 °C for 6 h. The mixture was allowed to reach room temperature under stirring overnight, and the resulting brown colored suspension was concentrated under vacuum and dialyzed against bidistilled water. To replace the I^-^ counterions with Cl^-^ counterions, the obtained polymer was treated with an NaCl solution (10% *w/w*, room temperature, overnight) and lyophilized. The dried polymer is stable at room temperature until the time of use and no further purification is needed. TMC was characterized by Proton Magnetic Resonance (^1^H-NMR) spectroscopy to assess the degree of quaternization. TMC samples were prepared solubilizing freeze-dried TMC in D_2_O (5 mg/1 mL). Spectra were determined on a Varian INOVA500 (Palo Alto, CA, USA) spectrometer at 27 °C and were recorded with the water suppression technique. Chemical shifts are expressed as δ value.

### 2.3. NP Preparation and Characterization

A water dispersion of SA (1 mg/mL) was added to a TMC dispersion (2 mg/mL) containing D9-PG or PG at different concentrations to obtain six different formulations (PGNP0, PGNP0.1, PGNP0.5, PGNP0.7, PGNP1, PGNP1D) as reported in Table 1. The mixture was kept under constant magnetic stirring (~1000 rpm) at room temperature for one hour and then sodium tripolyphosphate (TPP, 1 mg/mL) was added as cross-linking agent (~1000 rpm for 30 min). The volumetric ratio between TMC/SA/TPP was fixed to 6:1:2. A light opalescence revealed NP formation. PG concentrations in the final formulations were (0, 0.1, 0.5, 0.7, and 1 mg/mL).

The average diameter (nm ± SD) and polydispersity index (PDI) of all the formulations were determined by Photon Correlation Spectroscopy (PCS), using a Zetasizer nano-ZS (Malvern Instruments, Worcestershire, UK) Then, 0.2 mL of each formulation were diluted with bidistilled water up to 10 mL and backscattered by a helium-neon laser (633 nm) at an angle of 173° and a constant temperature of 25 °C.

ζ potential (ZP) was determined using the same instrument by means of the M3-PALS (Mixed Mode Measurement-Phase Analysis Light Scattering) technique, which measures the particle electrophoretic mobility. All the measurements were made in triplicate.

Morphology of freeze-dried nanoparticles was examined by scanning electron microscope (SEM).

The samples were fixed on a brass stub using carbon double-sided tape. Pictures were then taken at an excitation voltage of 20 kV using a Dual-beam Fei Nova Nano Lab 600 (Hillsboro, OR, USA), equipped with a high-brightness FEG source reaching resolutions up to 5 nm.

### 2.4. Stability Studies

Stability studies were carried out for 28 days on NP dispersions stored at 4 °C. At preselected time intervals, the refrigerated formulations were allowed to warm to room temperature under magnetic stirrer for 30 min and then the average diameter, polydispersity index, and ζ potential were determined as previously described. All the measurements were taken in triplicate.

### 2.5. Evaluation of Encapsulation Efficiency

The encapsulation efficiency of the PG containing NPs was estimated by means of an indirect method. NP dispersions were centrifuged at 15,000 rpm for 15 min at 4 °C (Scilogex mod. D3024R, Rocky Hill, CT, USA) and the PG amount in the supernatant was determined by High Performance Liquid Chromatography (HPLC). 

The encapsulation efficiency (EE%) was calculated using the following equation:

(1) $$\mathrm{EE}\%\;=\;\frac{{\mathrm{PG}}_{\mathrm{TOT}}-{\mathrm{PG}}_{\mathrm{SURN}}}{{\mathrm{PG}}_{\mathrm{TOT}}}\;\times \;100$$

In the calculation of EE% (Equation (1)), PG_TOT_ corresponds to the total amount of PG used in each sample preparation and PG_SURN_ is the amount of PG in the supernatant after centrifugation. All the measurements were made in triplicate.

PG content was quantified at 254 nm using a Flexar–Perkin Elmer HPLC equipped with a UV detector and a computer-integrating apparatus. The column was a C18 reversed-phase and the mobile phase was a mixture of acetic acid solution (0.5% *v/v*) and methanol (10:90 *v/v* and pH = 3.92) at a flow rate of 1.2 mL/min. The retention time was 3.75 min ± 0.01 s. A standard calibration curve was built up by using working standard solutions. Calibration graphs were plotted according to the linear regression analysis, which gave a correlation coefficient value (R^2^) of 0.999.

### 2.6. In Vitro Drug Release Studies

In vitro PG release studies were performed using Franz cells. A dialysis membrane (Spectra/Por^®^ Dialysis Membrane MWCO: 12–14 kD) was previously hydrated with bidistilled water for 24 h and then fixed between the two compartments of Franz cells. The receiving compartment was filled with a water/ethanol solution (40:60 *v/v*) maintained under constant agitation and at controlled temperature (37 °C). In the donor compartment, 500 µL of the samples (PGNP0.1, PGNP0.5, PGNP0.7, PGNP1) were deposited. At preselected intervals (15, 30, 45, 60, 120, 180, 240, and 300 min), the total content of the receiving compartment (~6.5 mL) was withdrawn, replaced with pre-thermostated, fresh water/ethanol solution, and analyzed by HPLC for PG content. All the analyses were performed in triplicate.

### 2.7. In Vitro Biocompatibility

The RPMI 2650 human nasal septum carcinoma cell line (Sigma Aldrich, Milan, Italy) was grown as a monolayer in 75 cm^2^ flasks, and incubated in 100% humidity and 5% CO_2_ at 37 °C, using RPMI1640 supplemented with fetal bovine serum, penicillin/streptomycin, and fungizone as culture medium. For toxicity studies, cells were seeded into 96-well plates (7.5 × 103 cells/well) and, after 24 h, were treated for 24 h with PGNP1D-deuterated PG-loaded nanoparticles and related unloaded formulations at different dilutions corresponding to 0.5, 5, 15, and 40 µg/mL of PG. After incubation, cells were washed three times with fresh medium and their viability was determined by the (3(4,5-dimethylthiazolyl-2)-2,5-diphenyltetrazolium bromide) colorimetric assay (MTT), adding 200 µL of MTT reagent (0.5 mg/mL in PBS) to each well. After 2–3 h, the formed formazan crystals were dissolved in DMSO and their concentration was spectrophotometrically quantified at 570 nm with a microplate reader (Synergy 4, Reader BioTek Instruments, AHSI S.P.A, Bernareggio, Monza and Brianza, Italy). All experiments were repeated at least three times. Results are shown as percent of cell viability in comparison with non-treated control cells (100% viability).

### 2.8. In Vivo Studies

PGNP1D nanoparticle formulation, containing 1 mg/mL of D9-PG (Table 1), was tested on male Sprague–Dawley rats (*n* = 16, weight range 270–300 g). Rats were immobilized in a plexiglass restrainer and exposed to PGNP1D or empty NPs by inhalation via an aerosol apparatus (Nebula M2000, Air Liquide Medical Systems, Bovezzo, Italy).

The rate of flow of the aerosol apparatus was about 0.3 mL/min. The administered dose was approximatively 1 mg/kg per minute or 0.3 mg per animal per minute, since the PG concentration of the PGNP1D nanoparticle formulation is 1 mg/mL. At 15 min, 30 min, 1 h, and 2 h after continuous inhalation, rats were deeply anesthetized and sacrificed by decapitation. Brains and blood were rapidly collected and processed to determine the amount of D9-PG.

All experimental procedures met the guidelines and protocols approved by the European Community (2010/63 UE L 276 20/10/2010) and by the Ethical Commission for Animal Care and Use at the University of Cagliari and Italian Ministry of Health (pr. # 1293/2015-PR, 18/12/2015).

### 2.9. Progesterone and Deuterated Progesterone (D9-PG) in Plasma and Brain GC-MS Analysis

Quantities of D9-PG in plasma and brain tissue was determined by GC-MS analysis. Plasma samples were initially thawed on ice, and then 200 µL of plasma were withdrawn and extracted with 250 µL of an ice-cold mixture of methanol/water (3:1 *v/v*). Samples were then loaded on C18 columns (Agilent Technologies, Palo Alto, CA, USA), which were previously activated with 2 mL of methanol and 2 mL of bidistilled water. One mL of methanol was added to each SPE column and recovered. The purified extract was then evaporated to dryness using a gentle nitrogen stream. 

Brain samples were initially weighed and thawed on ice. Then, 2 mL of a methanol/acetic acid (99:1 *v/v*) solution were added to each sample, which was homogenated with a Potter-Elvehjem PTFE pestle and glass tube homogenizer and left overnight. Samples were then centrifugated for 15 min at 4000 rpm and recovered. The pellet was dissolved and extracted again using 0.5 mL of the methanol/acetic acid solution. After centrifugation of the latter obtained solution, the two extracts were combined and evaporated to dryness. Brain and plasma samples were finally derivatized using 100 µL heptafluorobutyric acid (HFBA) and 200 µL acetone. After 30 min at 35 °C, 600 µL hexane were added before GC-MS analysis.

The derivatized samples were analyzed with a Hewlett Packard 6850 Gas Chromatograph, 5973 mass selective detector, and 7683B series injector (Agilent Technologies, Palo Alto, CA, USA), using helium as the carrier gas at 1.0 mL min^−1^ flow. One μL of samples was injected in the split-less mode and resolved on a 30 m × 0.25 mm × 0.25 μm DB-5MS column (Agilent Technologies, Palo Alto, CA, USA). Inlet, interface, and ion source temperatures were 250, 250, and 230 °C, respectively. Oven starting temperature was set to 50 °C, and the final temperature to 230 °C with a heating rate of 5 °C/min for 36 min and then for 2 min at constant temperature. GC-MS mass spectra were recorded in the selected ion monitoring mode (SIM) recording the 510, 495, 425 *m/z* ions for PG and deuterated PG.

### 2.10. Statistical Analysis of Data

Results are expressed as the mean ± standard deviation and significance was tested at the 0.01 or 0.05 level of probability (*p*). For size, zeta potential, drug accumulation, and cytotoxicity, analysis of variance (one way-ANOVA) followed by post-hoc Bonferroni correction were used to substantiate statistical differences between groups using XLSTAT for Excel.

## 3. Results and Discussion

### 3.1. TMC Synthesis

In this study PG-loaded hydrogel NPs were prepared by ionotropic gelation technique, using a mixture of TMC/SA and TPP as cross-linker. TMC was synthesized as already reported [21]. The procedure was reproducible and allowed us to obtain TMC with a high degree of quaternization (DQ = 70%), simply extending the reaction time up to seven hours. By means of this procedure, we were able to reduce the methylating agent (CH_3_I) amount and, as demonstrated by ^1^H-NMR data, avoid the formation of undesirable side products (such as TMC with a high degree of methoxylation in position 3 and 6 of the glucosamine ring), which decrease the polymer water solubility. Besides, we avoided the use of organic solvents in purification steps.

### 3.2. Preparation and Characterization of Nanoparticles

The NP composition was selected based on results obtained in an our previous preformulation study in which the effect of both polymers (TMC and SA) and cross-linker (TPP) ratio was deeply investigated [21]. In particular, in this work the volumetric ratio of TMC, SA, and TPP solution was kept constant (6:1:2). Moreover, the amount of TMC was kept higher than that of SA in order to obtain NPs with positive surface charge, useful to promote the interaction with negative sialic residues of the nasal mucosa. Starting from the above reported composition, we prepared five PG NP formulations (PGNP 0, PGNP0.1, PGNP0.5, PGNP0.7, and PGNP1) loaded with different drug concentrations (0, 0.1, 0.5, 0.7, and 1 mg/mL, respectively). Moreover, a D9-PG-loaded NP dispersion was also prepared (PGNP1D, 1 mg/mL). Deuterated drug-loaded NPs have been used for in vivo studies in order to distinguish endogenous and administered PG after the inhalation experiment. Empty (PGNP0) and PG or D9-PG-containing NPs (PGNP0.1, PGNP0.5, PGNP0.7, PGNP1, PGNP1D) were fully characterized by average diameter (nm ± SD), polydispersity index (PDI), ζ potential (ZP), and encapsulation efficiency (EE%) (Table 1). As shown in Table 1, the incorporation of PG or D9-PG led only to a slight variation of the NP mean diameter, PDI, and ζ potential values when compared to the unloaded NP. Indeed, all formulations (unloaded and loaded) showed a mean diameter ranging from 200 nm and 236 nm and a polydispersity index always smaller than 0.23, thus indicating a fairly narrow size distribution.

The zeta potential values, all positive and greater than 28 mV, should ensure NP stability against aggregation phenomena. In general, all loaded formulations showed a high EE%. The amount of PG used for the preparation of the different formulations seems to have only a small influence on the drug encapsulation efficiency (EE%). Indeed, PGNP0.1, prepared with the smallest PG concentration (0.1 mg/mL), showed the highest EE% (95%), but PGNP1, with the highest PG concentration (1 mg/mL) can also incorporate a very high amount of drug (EE% 83).

**Figure 1:**
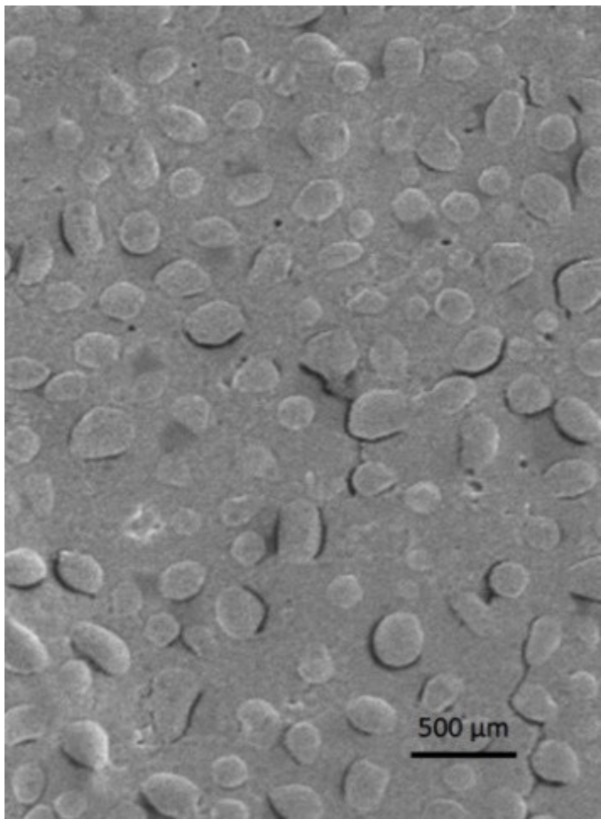
Scanning electron microscopy image of freeze-dried PGNP1D nanoparticles.

NP morphology was evaluated via scanning electron microscopy. In Figure 1 an SEM image of freeze-dried PGNP1D nanoparticles is reported. As can be seen, freeze-dried PG-loaded NPs have a regular and rounded shape. Moreover, SEM analyses confirmed the fact that nanoparticles with a mean diameter comparable with that measured by photon correlation spectroscopy are still present in the lyophilized samples.

### 3.3. Stability of NPs

**Figure 2:**
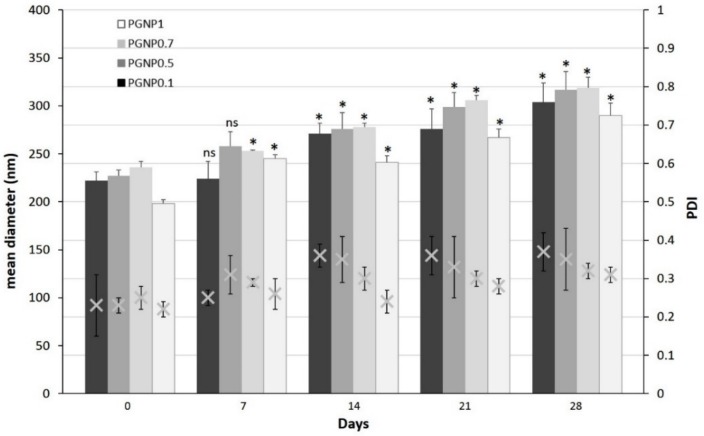
Variation of mean diameter and polydispersity index (PDI) of PG-loaded nanoparticles during 28 days of storage at 4 °C. Results are expressed as means of three independent measurements ± standard deviations. For each formulation, statistical analysis was performed comparing the mean diameter value at each time point with respect to that at time zero. * = different from day 0 (*p* ≤ 0.05), ns indicates not significant data (*p* > 0.05).

**Figure 3:**
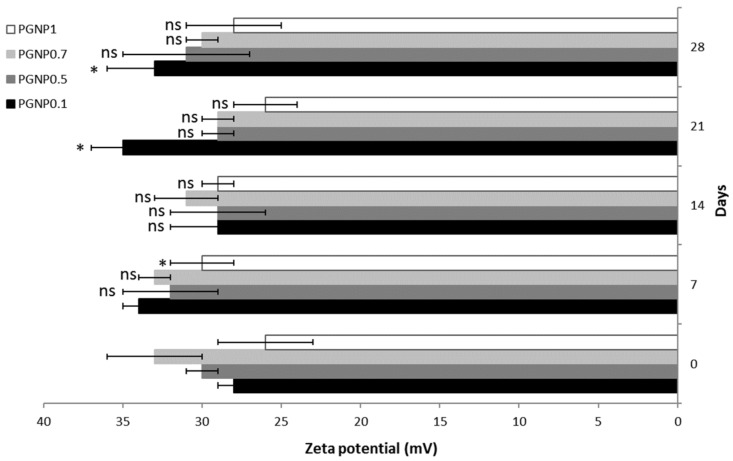
Zeta potential of PG-loaded nanoparticles during 28 days of storage at 4 °C. Results are expressed as means of three independent measurements ± standard deviations. For each formulation, statistical analysis was performed comparing the zeta potential value at each time point with respect to that at time zero. * = different from day 0 (*p* ≤ 0.05), ns indicates not significant data (*p* > 0.05).

Stability studies were performed for 4 weeks, monitoring the variations in size, PDI, and ζ potential of NPs stored at 4 °C (Figure 2 and Figure 3). As shown in Figure 2, the NP mean diameter growth over the studied period was moderate (ranging around 30%). Indeed, all formulations showed a mean diameter of approximately 300 nm after 28 days of storage at 4 °C and, in particular, the smallest size (~290 nm) was measured for PGNP1. Also, polydispersity index values increased after 28 days, thus confirming the fact that NP weak aggregation phenomena occur during storage. Once again, the formulation PGNP1 showed the smallest PDI (nearby 0.30). As expected, zeta potential remains positive for all NP, and almost stable in a narrow range (26–35 mV) over the monitored period (Figure 3).

### 3.4. PG Release from NPs

**Figure 4:**
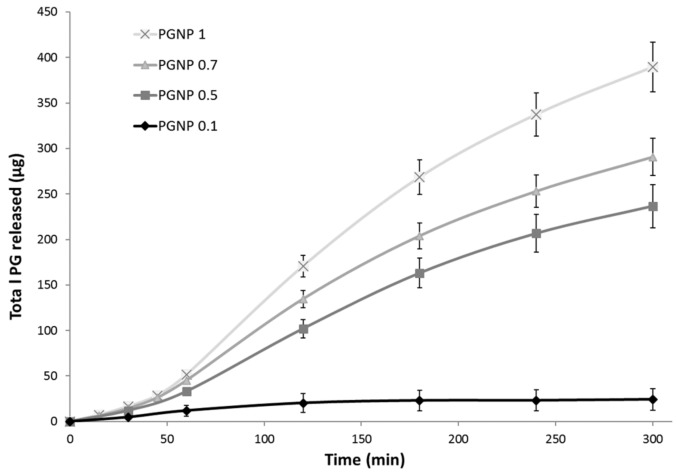
In vitro PG release in water/ethanol (40:60 *v/v*) solution from PG-loaded nanoparticles.

The PG release from hydrogel NPs was studied over a period of 5 h in a water/ethanol (40:60 *v/v*) solution to ensure sink conditions. To this aim, 500 µL of each samples (PGNP0.1, PGNP0.5, PGNP0.7, PGNP1) were deposited in the Franz cell donor compartment while the receiving compartment was filled with a water/ethanol solution (40:60 *v/v*) maintained at 37 °C and under constant agitation. Results reported in Figure 4 show no burst release for all formulations, thus demonstrating that PG is effectively incorporated into the hydrogel nanoparticles matrix. The PG release profile from formulations PGNP1, PGNP0.7, and PGNP0.5 showed a similar trend, with the release rate directly related to the loaded drug concentration. As for PG release from NPs with the lowest PG concentration, namely PGNP0.1, the rate is significantly the lowest (50.52%). This behavior suggested that the main mechanism by which PG is released from NPs should be a concentration-dependent diffusion process rather than a consequence of matrix hydrogel erosion.

Starting from the release results and taking also into account stability data, we decided to perform the in vivo studies using PGNP1 but loaded with deuterated PG (D9-PG), namely the PGNP1D formulation (Table 1).

### 3.5. In Vitro Biocompatibility 

**Figure 5:**
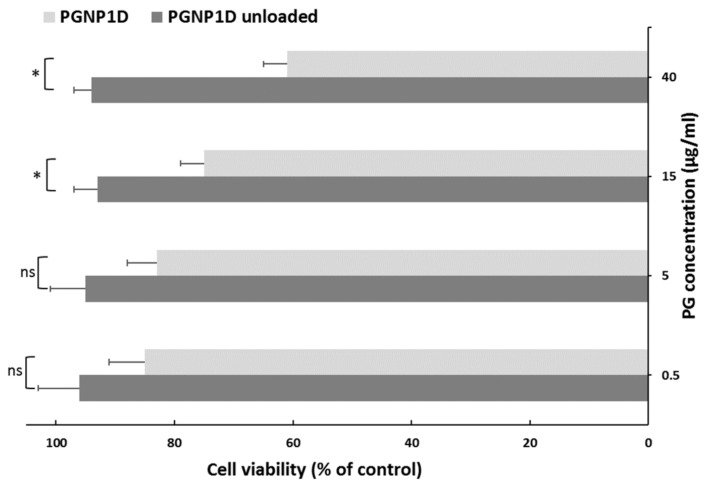
In vitro cytotoxicity studies of PGNP1D and relative unloaded formulations at different dilutions on the human nasal cell line RPMI 2650. The cell viability is recorded as percentage cell viability in comparison with non-treated control cells (100% viability). Results are represented as mean ± SD from three independent experiments. The asterisks indicate statistically significant data (* = *p* ≤ 0.05), ns indicates not significant data (*p* > 0.05).

To evaluate the biocompatibility of both PGNP1D-unloaded and deuterated PG-loaded nanoparticles, an in vitro cytotoxicity study was performed using the human nasal septum carcinoma cell line treated with different PG concentrations (0.5, 5, 15, and 40 µg/mL) for 24 h. As can be seen in Figure 5, the unloaded formulation did not induce any toxic effect on RPMI 2650 cells exposed for 24 h up to the highest tested dose. However, the presence of PG reduces the cell viability in an apparently dose-dependent manner. Indeed, at the dose of 40 µg/mL, a 61% cell viability, when compared to non-treated control cells, was observed. These results confirmed data already reported in the literature concerning the apoptosis effect produced by PG at high doses and prolonged exposition times on cells [24].

### 3.6. In Vivo Studies

**Figure 6:**
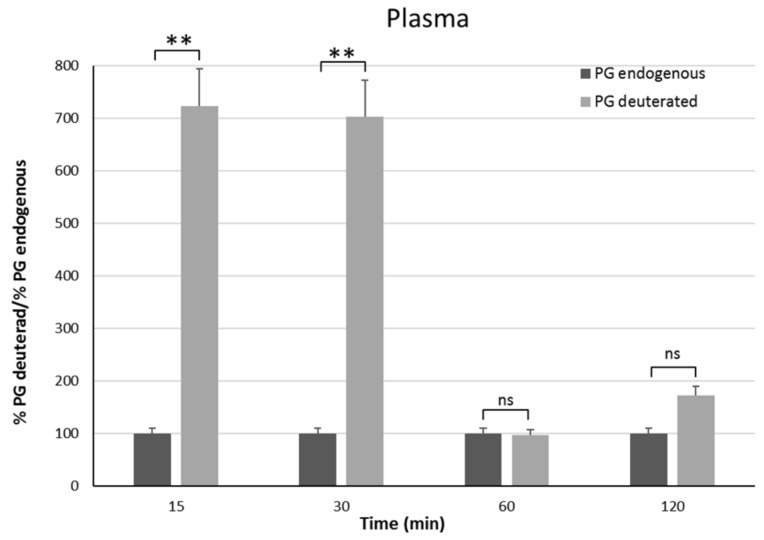
Plasma endogenous and deuterated PG detected on male Sprague–Dawley rats 15 min, 30 min, 1 h, and 2 h after continuous PGNP1D inhalation. The asterisks indicate statistically significant data (** = *p* ≤ 0.01), ns indicates not significant data (*p* > 0.05).

**Figure 7:**
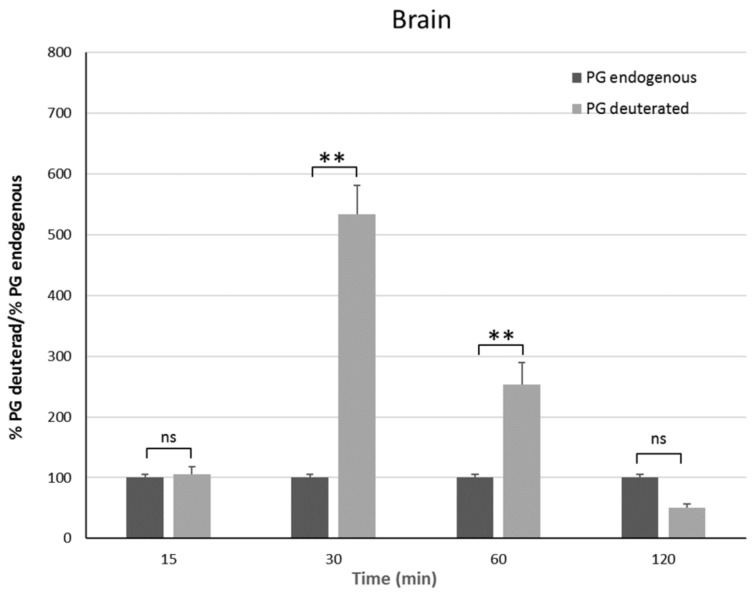
Brain endogenous and deuterated PG detected on male Sprague–Dawley mice 15 min, 30 min, 1 h, and 2 h after continuous PGNP1D inhalation. The asterisks indicate statistically significant data (** = *p* ≤ 0.01), ns indicates not significant data (*p* > 0.05).

**Figure 8:**
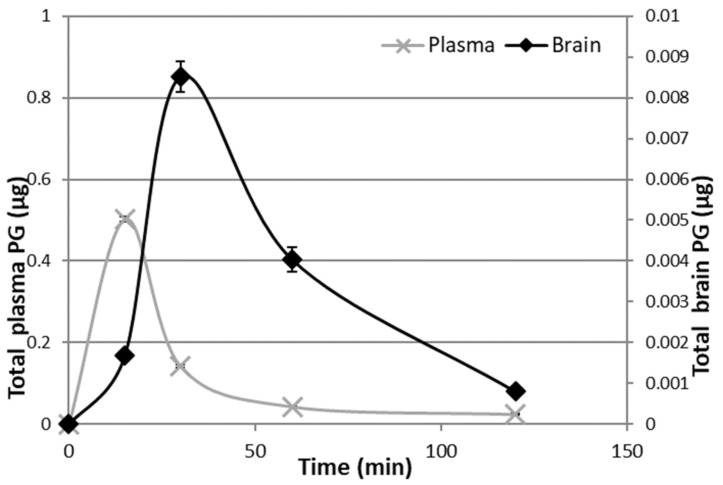
Deuterated PG in plasma and brain 15 min, 30 min, 1 h, and 2 h after continuous PGNP1D inhalation.

In vivo studies have been carried out on male rats in order to have an animal model with levels of PG as constant as possible. Indeed, the level of endogenous PG in female rats depends on the estrus phase, and thus it is much more variable than in males [25]. To discriminate endogenous PG from exogenous PG, we administered the PGNP1D or empty nanoparticles via aerosol, and we measured plasma and brain levels of both D-9-progesterone and endogenous progesterone at progressive time points (Figure 6 and Figure 7). Results showed that deuterated PG reached a plasma concentration six-fold higher than endogenous PG as soon as 15 min after nanoparticles inhalation, which lasted for 30 min. Plasma levels of deuterated PG decreased to values close to that of endogenous PG 60 min after inhalation (Figure 6). Analysis of brain tissue showed that deuterated PG increased to a concentration significantly higher than endogenous PG when measured 30 min and 60 min after inhalation, and it returned to endogenous PG level 120 min after inhalation (Figure 7). In Figure 8, the total amount of deuterated PG in plasma and brain tissue at preselected time points up to 120 min is reported. As can be clearly observed from the graph, the maximum amount of D9-PG is detected in plasma after 15 min (~0.5 µg) and it is significantly higher than the maximum detected in brain tissue after 30 min (~0.0083 µg). Therefore, in vivo results demonstrate that the nanoparticles used in the present study efficiently carried PG into the brain, even though we do not have any evidence concerning the pathway by which PG reaches the brain tissue. NPs may allow PG to directly reach the brain parenchyma via the nose-to-brain olfactory pathway, bypassing the BBB. However, obtained results seem also to indicate that PG released from NPs diffuses from the nasal mucosa into the blood stream, from where it may pass the blood–brain barrier according to its lipophilic nature [26].

## 4. Conclusions

In this work, NPs loaded with different PG concentrations were obtained from trimethyl chitosan and sodium alginate cross-linked with sodium tripolyphosphate. Among the various prepared NPs, characterization studies allowed us to select the best formulation (PGNP1) in terms of size, stability, and drug release profile. These NPs have been loaded with deuterated PG and tested in vivo on Sprague–Dawley male rats. In vivo results demonstrate that the selected nanoparticles could be an efficient carrier for PG brain delivery useful in the treatment of different neurodegenerative disorders. Further research will be carried out in order to identify the main route involved in PG delivery to the brain tissue.

## Figures and Tables

**Table 1:** Composition mean diameter (nm), polydispersity index (PDI), and zeta potential (ZP, mV) of freshly prepared progesterone (PG)-loaded nanoparticles. Results are expressed as means of three independent measurements ± standard deviations. EE%: encapsulation efficiency. PGNP0, PGNP0.1, PGNP0.5, PGNP0.7, and PGNP1: PG NP formulations loaded with drug concentrations of 0, 0.1, 0.5, 0.7, and 1 mg/mL, respectively. NP: nanoparticle.

Sample	PG Concentration (mg/mL)	D9-PG Conccentration (mg/mL)	Mean Diameter (nm ± SD)	PDI (±SD)	ZP (mV ± SD)	EE%
PGNP0	-	-	208 ± 5	0.19 ± 0.01	+28 ± 2	/
PGNP0.1	0.1	-	222 ± 9	0.23 ± 0.08	+28 ± 1	95 ± 3
PGNP0.5	0.5	-	227 ± 6	0.23 ± 0.04	+30 ± 1	88 ± 5
PGNP0.7	0.7	-	236 ± 6	0.25 ± 0.03	+31 ± 2	85 ± 5
PGNP1	1	-	198 ± 4	0.22 ± 0.02	+29 ± 1	83 ± 2
PGNP1D	-	1	204 ± 5	0.25 ± 0.03	+27 ± 1	80 ± 1
